# Supplementary material for: Beyond benchmarking and towards predictive models of dataset-specific single-cell RNA-seq pipeline performance
Source: Genome Biol. 2024 Jun 17;25:159. doi: 10.1186/s13059-024-03304-9 (PMC11184819; doi:10.1186/s13059-024-03304-9)
Supplement: Supplementary file 1 — Additional file 1. All supplementary figures for this study. [file 13059_2024_3304_MOESM1_ESM.docx]

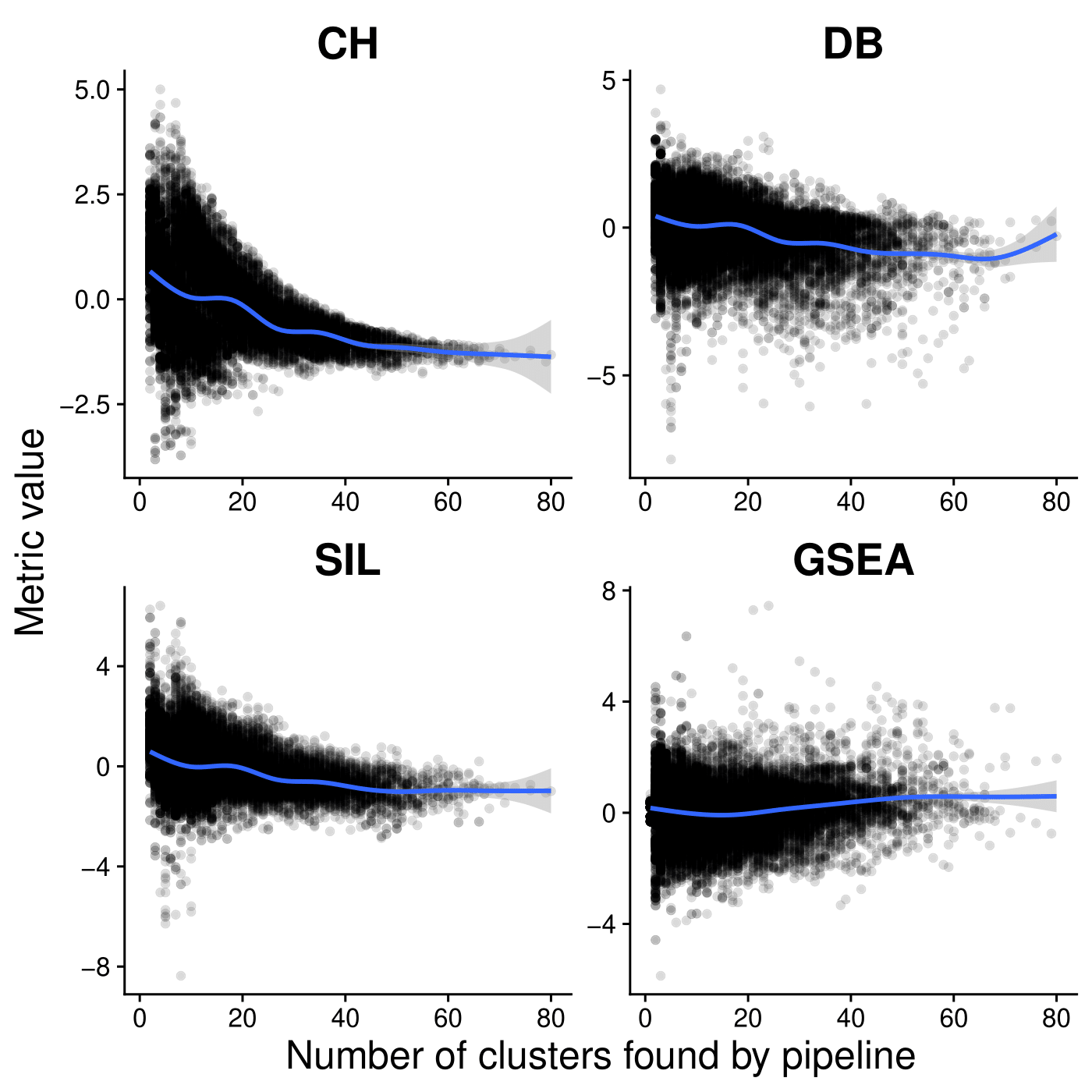


**Fig. S1:** Correlation of the four unsupervised metrics with the number of clusters found by a given pipeline across all datasets.


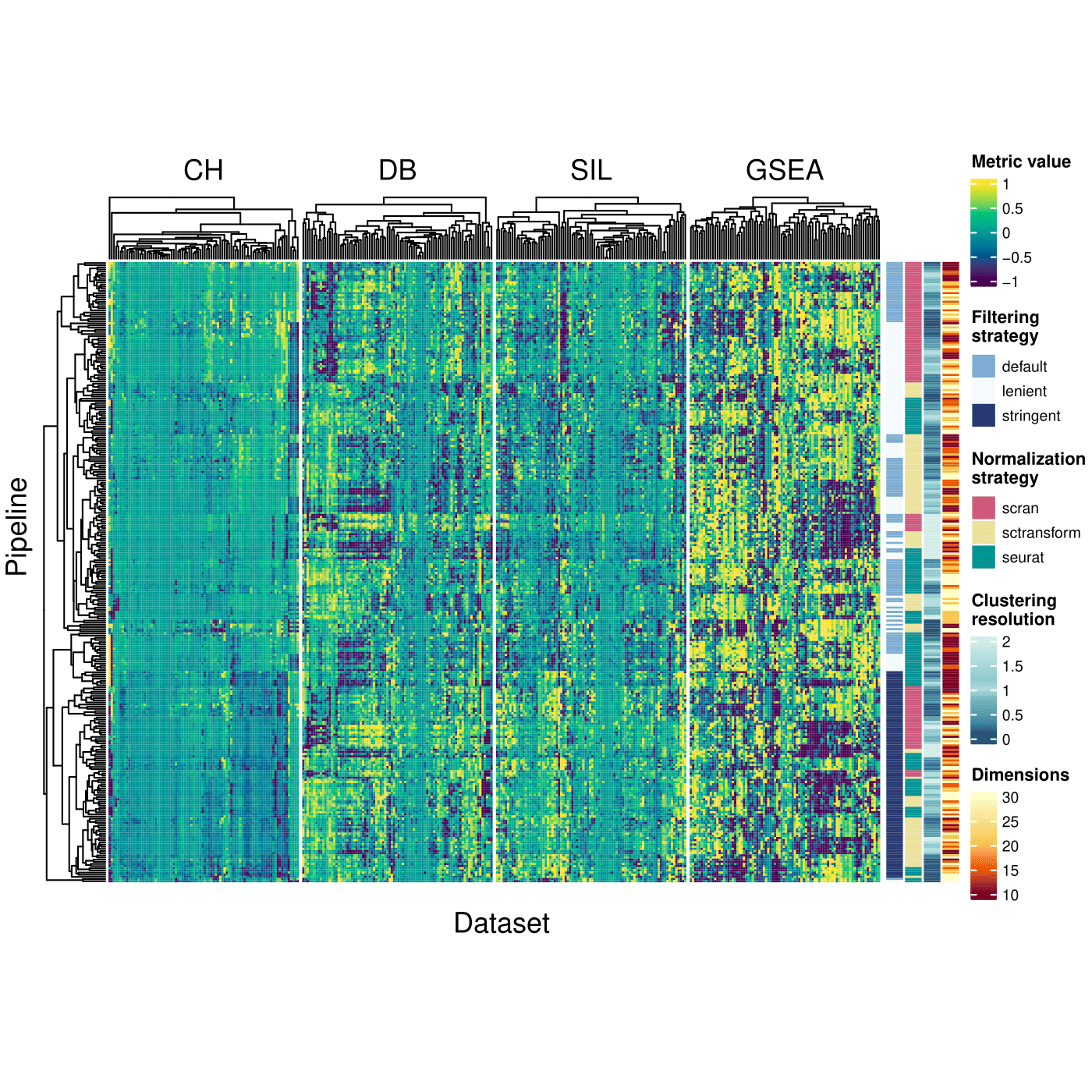


**Fig. S2**: Performance of each pipeline across datasets when the effect of the number of clusters has been removed via nonparametric regression.

**Fig. S3**: Every pipeline visualized by the number of times it had the highest metric value over all datasets. No single pipeline achieved the best performance across all datasets.


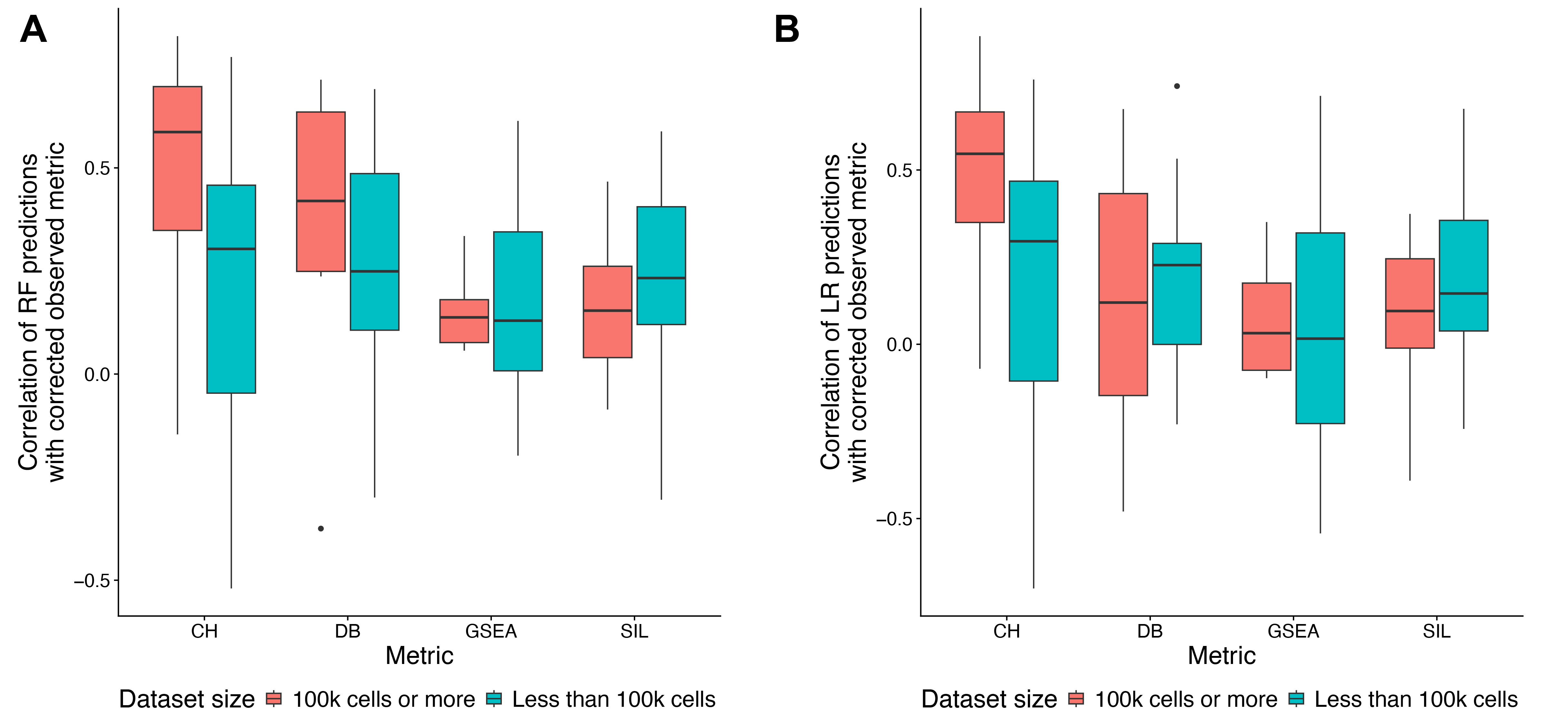
**Fig. S4:** Correlation between true and predicted metric values for 192 pipelines on 6 datasets with >100k cells.


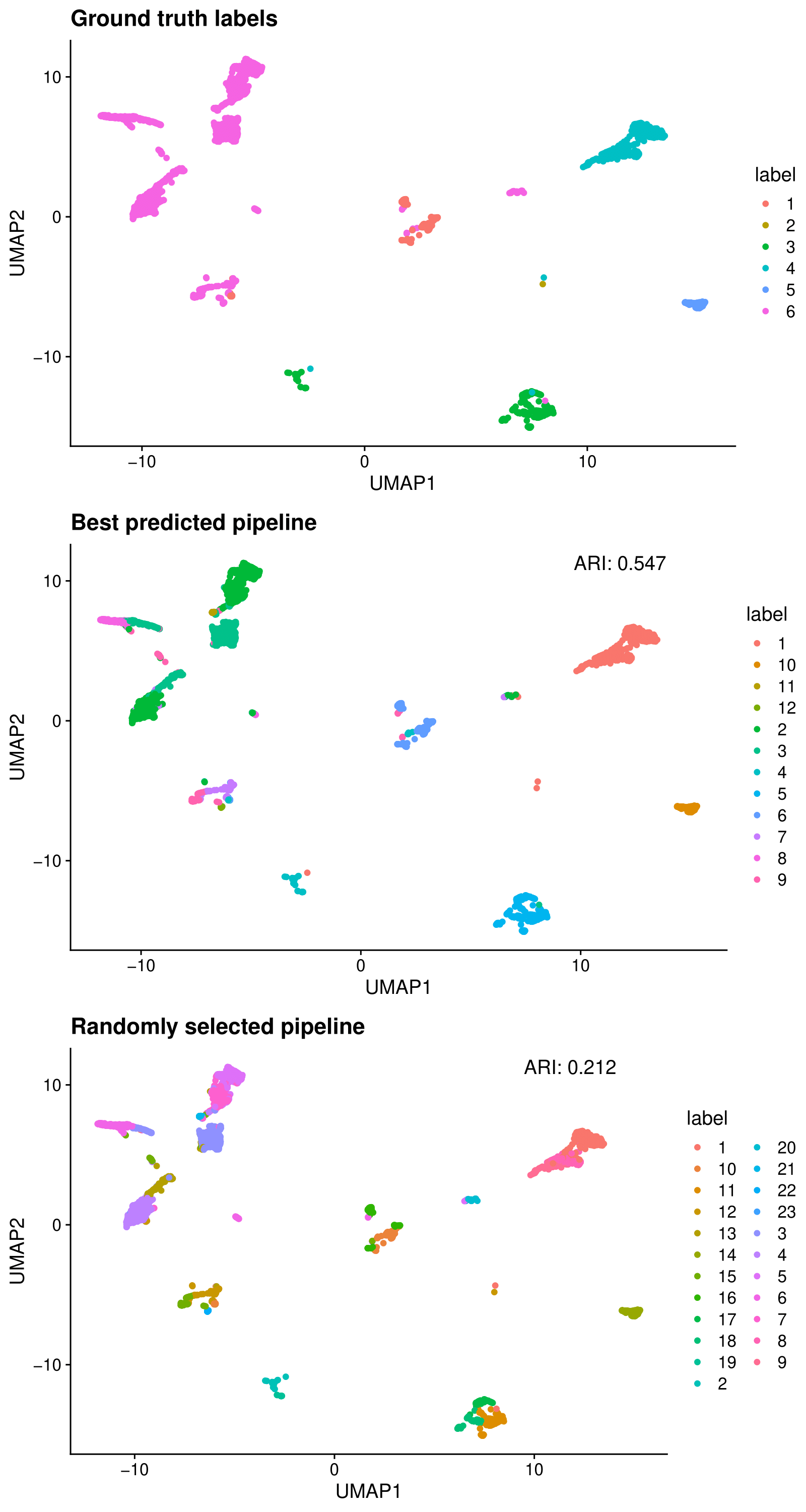


**Fig. S5:** UMAP on first 50 principal components of an example dataset (EBI ID: E-MTAB-9221) with expert annotations, coloured by expert annotations (ground truth labels), labels from the best pipeline predicted by the RF with interaction terms model for SIL, and a randomly selected pipeline. UMAP computed on the first 50 PCs using scater.


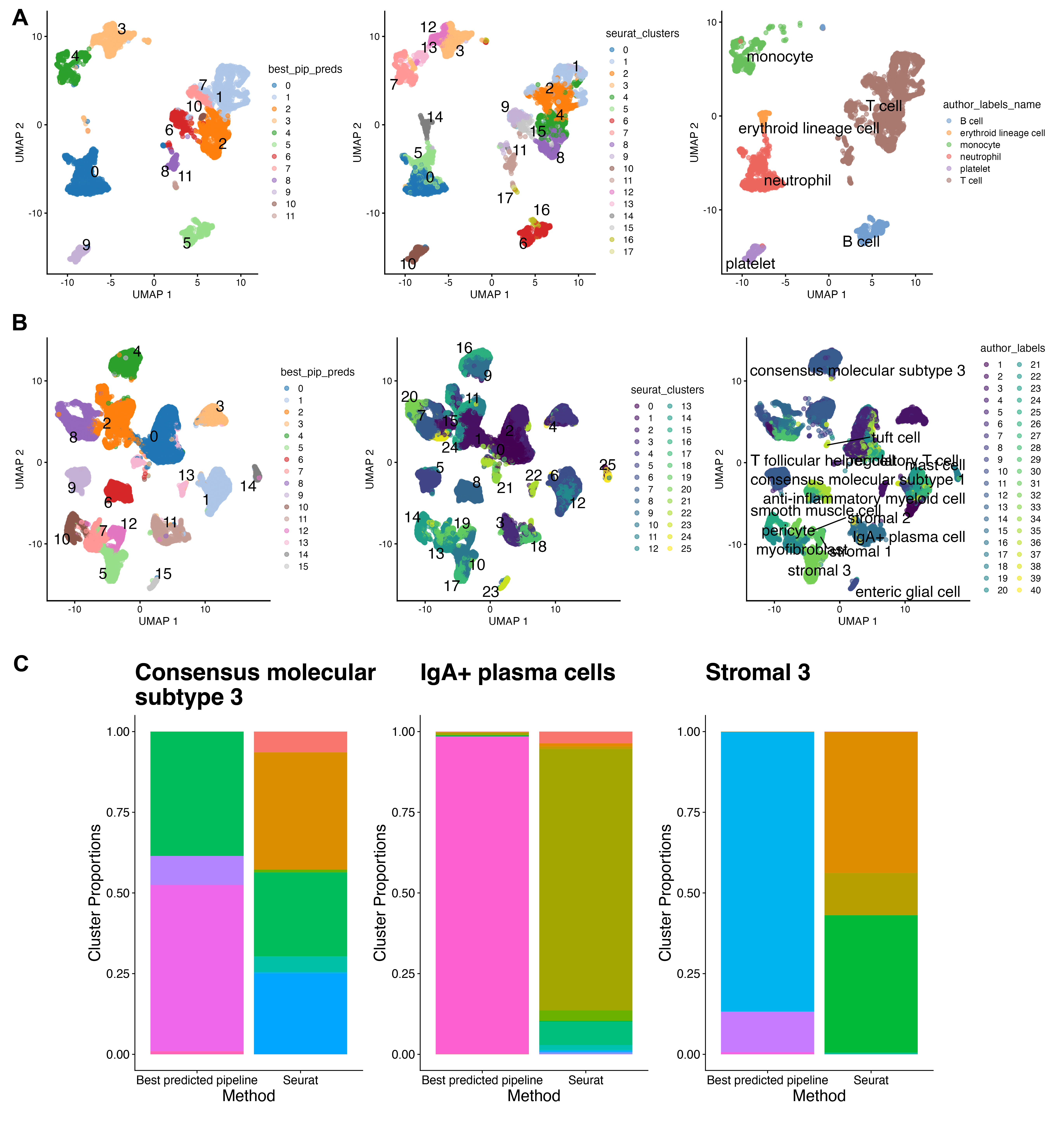


**Fig. S6: A** UMAP of dataset E-MTAB-9221 with cells coloured by clusters returned by best SIL predicted pipeline (left), Seurat defaults (middle), and author annotations (right). **B** As (**A**) for E-MTAB-8410. **C** The cluster proportions returned by best predicted pipeline and Seurat default pipeline for three representative annotated cell types in E-MTAB-8410. UMAP computed using Seurat’s default pipeline.


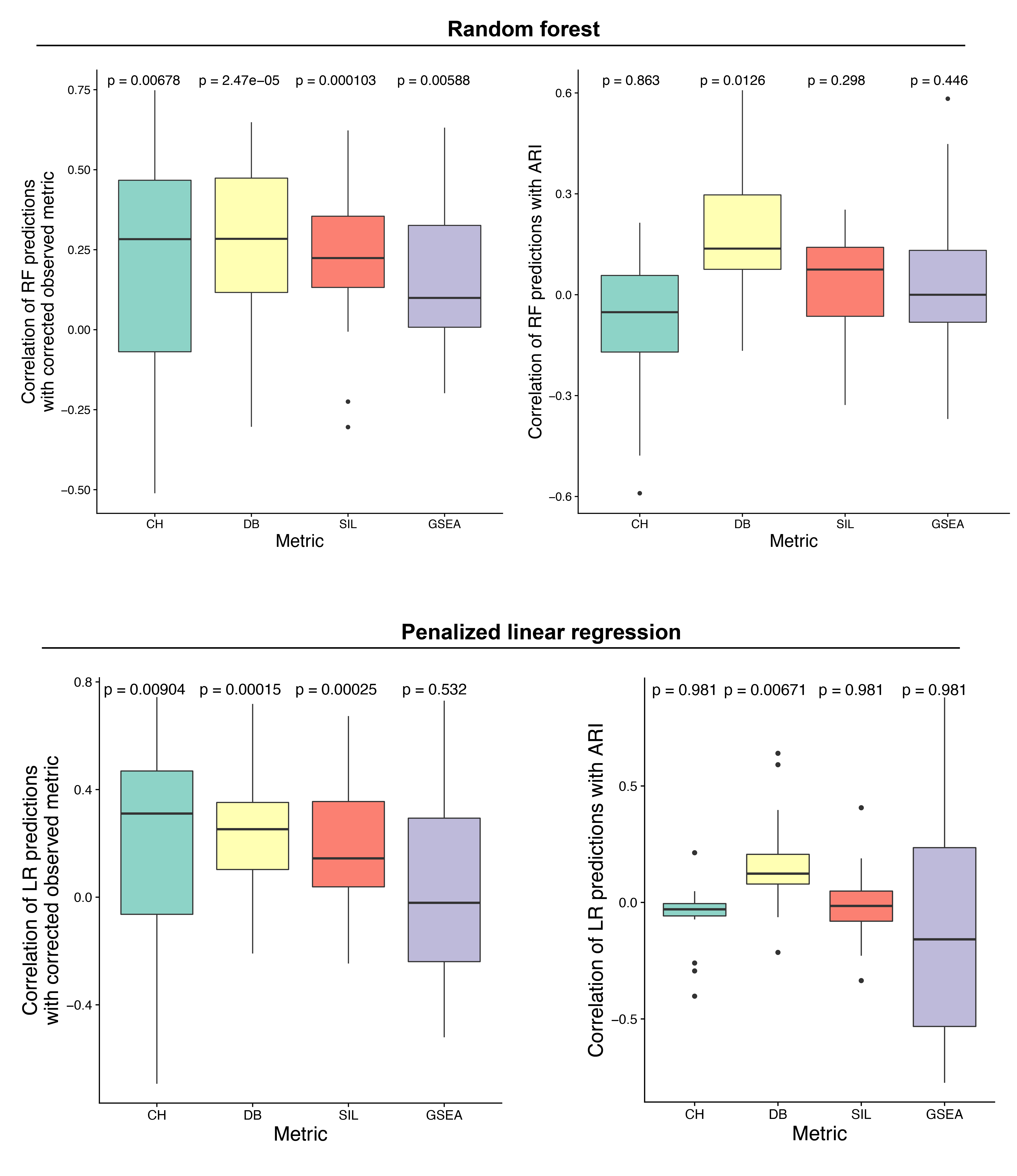


**Fig. S7:** Prediction performance of the different machine learning models when incorporating additional metadata features into dataset-specific pipeline performance modelling.


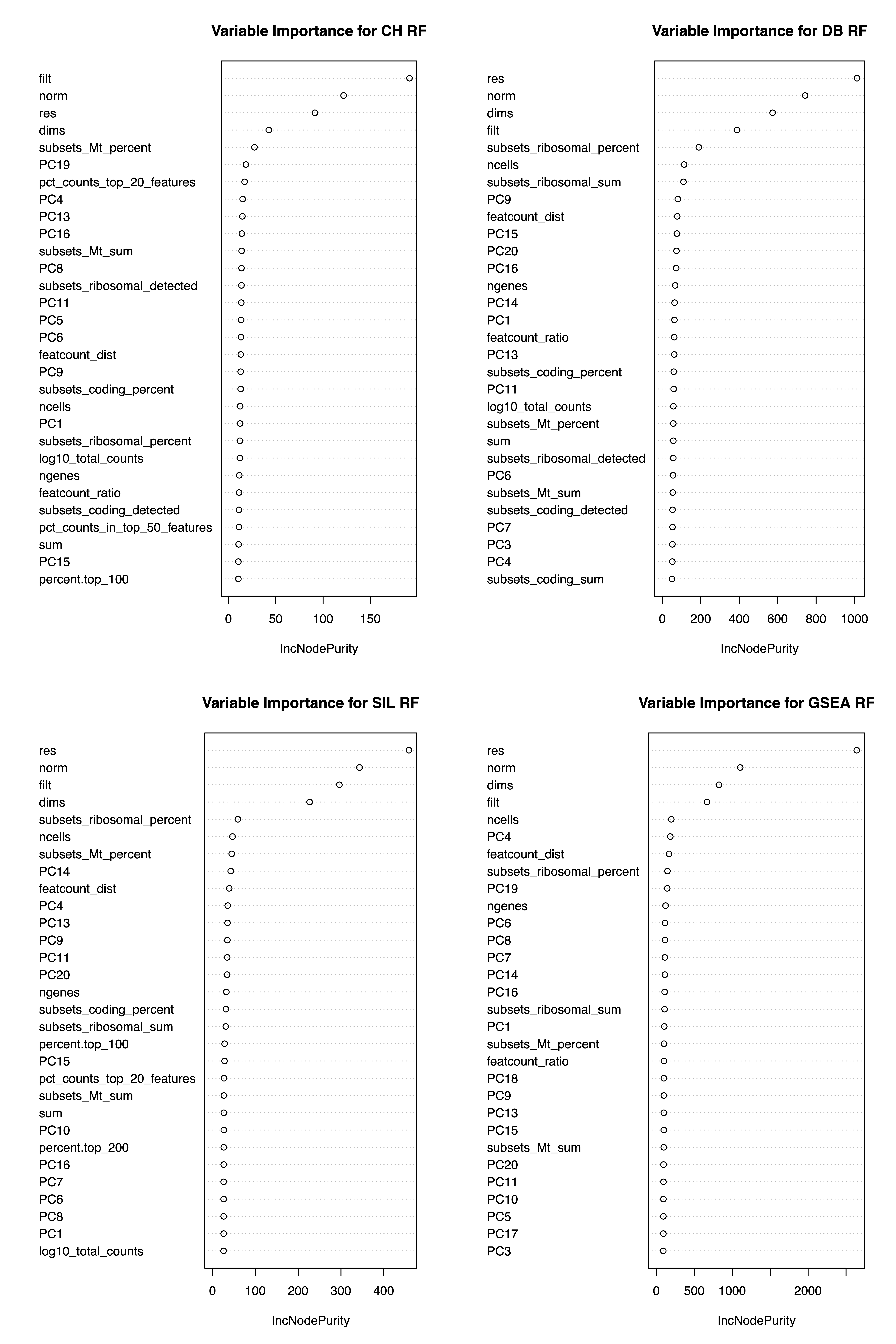


**Fig. S8:** Top most important features (as measured via IncNodePurity) for the random forest model (with full dataset-pipeline features).


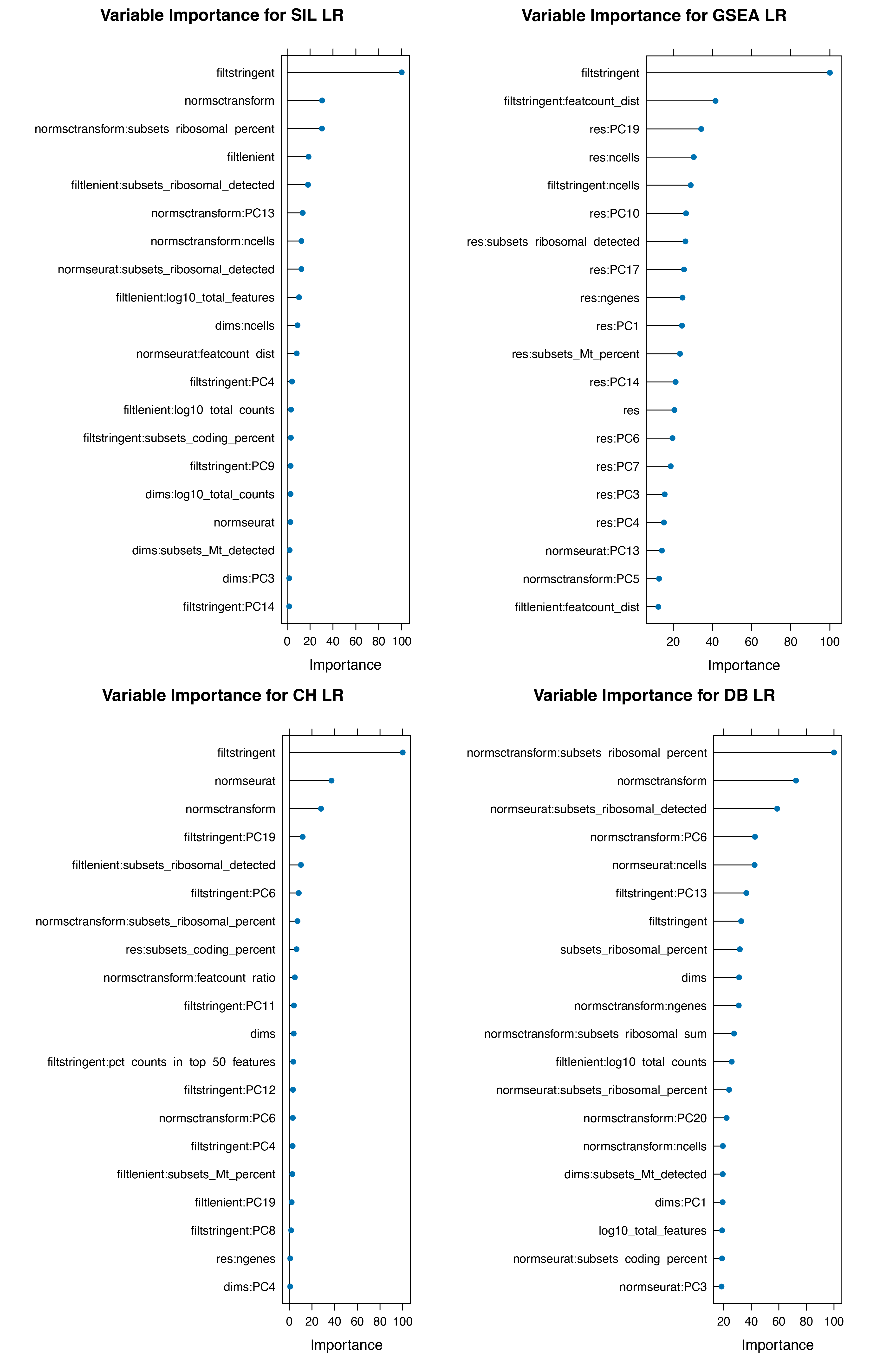


**Fig. S9:** Top most important features (as measured via absolute coefficient size) for the penalized linear regression model (with full dataset-pipeline features).
