# Supplementary material for: Beyond benchmarking and towards predictive models of dataset-specific single-cell RNA-seq pipeline performance
Source: Genome Biol. 2024 Jun 17;25:159. doi: 10.1186/s13059-024-03304-9 (PMC11184819; doi:10.1186/s13059-024-03304-9)
Supplement: Supplementary file 2 — Additional file 2. All supplementary tables for this study. [file 13059_2024_3304_MOESM2_ESM.docx]

| **EBI ID** | **Organism part** | **Number of cells** |
| --- | --- | --- |
| E-CURD-10 | kidney | 118 |
| E-CURD-11 | bronchiole, lung, middle lobe of right lung | 176 |
| E-CURD-55 | blood | 97588 |
| E-CURD-6 | bone marrow | 1024 |
| E-CURD-7 | breast epithelium | 809 |
| E-CURD-85 | synovial membrane, blood, synovial fluid | 77035 |
| E-ENAD-17 | brain | 96 |
| E-ENAD-20 | tumor xenograft | 674 |
| E-ENAD-21 | breast | 809 |
| E-ENAD-27 | islet of Langerhans | 1145 |
| E-GEOD-100618 | blood | 415 |
| E-GEOD-106540 | blood | 2244 |
| E-GEOD-109979 | embryo | 329 |
| E-GEOD-110499 | bone marrow | 173 |
| E-GEOD-111727 | blood | 342 |
| E-GEOD-114530 | kidney | 20426 |
| E-GEOD-124263 | testis | 51504 |
| E-GEOD-124472 | inner cell mass, juxtamedullary cortex, outer cortex of kidney | 16094 |
| E-GEOD-124858 | bone marrow | 241 |
| E-GEOD-125970 | intestine | 18540 |
| E-GEOD-130148 | lung parenchyma | 4788 |
| E-GEOD-130473 | liver | 1307 |
| E-GEOD-134144 | testis | 31565 |
| E-GEOD-135922 | retina | 23280 |
| E-GEOD-137537 | retina | 7275 |
| E-GEOD-149689 | blood | 77690 |
| E-GEOD-36552 | embryo | 124 |
| E-GEOD-70580 | tonsil | 648 |
| E-GEOD-75140 | neocortex, inner cell mass, skin | 734 |
| E-GEOD-75367 | blood | 74 |
| E-GEOD-75688 | breast, lymph | 540 |
| E-GEOD-76312 | bone marrow | 2151 |
| E-GEOD-81383 | cell line | 226 |
| E-GEOD-81547 | islet of Langerhans | 2544 |
| E-GEOD-81608 | pancreatic islet | 1600 |
| E-GEOD-83139 | pancreatic islet | 635 |
| E-GEOD-84465 | brain | 3576 |
| E-GEOD-86618 | lung | 540 |
| E-GEOD-89232 | blood, umbilical cord blood | 957 |
| E-GEOD-93593 | embryo | 1733 |
| E-GEOD-98556 | inner cell mass, retina | 546 |
| E-GEOD-99795 | prostate | 144 |
| E-HCAD-10 | kidney | 33162 |
| E-HCAD-11 | colon | 23517 |
| E-HCAD-13 | skin epidermis | 6209 |
| E-HCAD-23 | decidua, chorion membrane | 12509 |
| E-HCAD-24 | chorion membrane, decidua | 17611 |
| E-HCAD-25 | cerebellar cortex, substania nigra | 22566 |
| E-HCAD-31 | pancreas | 22753 |
| E-HCAD-32 | blood, spleen, liver | 61144 |
| E-HCAD-35 | brain | 34348 |
| E-HCAD-36 | blood vessel | 59102 |
| E-HCAD-38 | epididymis | 11765 |
| E-HCAD-5 | skin of body | 23474 |
| E-HCAD-6 | bone marrow | 32445 |
| E-HCAD-8 | lung, bone marrow, lymph node, blood | 80524 |
| E-HCAD-9 | liver | 11265 |
| E-MTAB-4850 | blood | 63 |
| E-MTAB-5061 | pancreas | 3229 |
| E-MTAB-6108 | retina | 1449 |
| E-MTAB-6142 | adipose tissue | 96 |
| E-MTAB-6308 | lung | 77638 |
| E-MTAB-6379 | blood | 119 |
| E-MTAB-6386 | blood | 117 |
| E-MTAB-6505 | umbilical cord blood | 10316 |
| E-MTAB-6524 | skin | 10475 |
| E-MTAB-6653 | lung | 33209 |
| E-MTAB-6678 | decidua, blood | 7484 |
| E-MTAB-6819 | inner cell mass | 1344 |
| E-MTAB-6911 | dermis, blood | 332 |
| E-MTAB-7008 | blood | 1018 |
| E-MTAB-7037 | dermis | 590 |
| E-MTAB-7051 | dermis | 681 |
| E-MTAB-7052 | dermis | 1920 |
| E-MTAB-7249 | ascitic fluid | 192 |
| E-MTAB-7303 | skin | 123 |
| E-MTAB-7316 | retinal neural layer | 20520 |
| E-MTAB-7381 | tonsil | 352 |
| E-MTAB-7606 | blood | 209 |
| E-MTAB-8271 | blood | 2720 |
| E-MTAB-8410 | caecum, rectosigmoid junction, sigmoid colon, colon, ascending colon | 60383 |
| E-MTAB-8495 | common bile duct, gall bladder, intrahepatic bile duct | 82433 |
| E-MTAB-8559 | peritoneal fluid | 20321 |
| E-MTAB-8911 | blood | 18508 |
| E-MTAB-9067 | bone marrow, liver | 5625 |
| E-MTAB-9221 | blood | 6178 |
| E-CURD-122 | cross-tissue immune cells | 115,203 |

**Table S1:** scRNA-seq datasets used in this study. All datasets are publicly available through the EBI Single Cell Expression Atlas.

| **Name** | **Description** | **Feature encoding for ML models** |
| --- | --- | --- |
| sum | the sum of counts for each cell | Numeric |
| detected | Number of genes detected per cell (above a user-specified threshold) | Numeric |
| percent.top_100 | percentage of counts assigned to the top 100 most highly expressed genes per cell | Numeric |
| percent.top_200 | percentage of counts assigned to the top 200 most highly expressed genes per cell | Numeric |
| subsets_Mt_sum | Total sum of mitochondrial counts for each cell | Numeric |
| subsets_Mt_detected | Number of genes detected per cell assigned to mitochondrial counts | Numeric |
| subsets_Mt_percent | percentage of each cell’s count sum assigned to mitochondrial counts | Numeric |
| subsets_coding_sum | Total sum of coding counts for each cell | Numeric |
| subsets_coding_detected | Number of genes detected per cell assigned to coding counts | Numeric |
| subsets_coding_percent | percentage of each cell’s count sum assigned to coding counts. | Numeric |
| subsets_ribosomal_sum | Total sum of ribosomal counts for each cell | Numeric |
| subsets_ribosomal_detected | Number of genes detected per cell assigned to ribosomal counts | Numeric |
| subsets_ribosomal_percent | percentage of each cell’s count sum assigned to ribosomal counts | Numeric |
| log10_total_features | log10(detected) | Numeric |
| log10_total_counts | log10(sum+1) | Numeric |
| featcount_ratio | log10_total_counts/log10_total_features | Numeric |
| featcount_dist | distance to expected ratio of log10 counts and features | Numeric |
| pct_counts_top_20_features | percentage of counts assigned to the top 20 most highly expressed genes per cell | Numeric |
| pct_counts_in_top_50_features | percentage of counts assigned to the top 50 most highly expressed genes per cell | Numeric |
| ncells | Total number of cells in dataset | Numeric |
| ngenes | Total number of genes in dataset | Numeric |

**Table S2:** Features computed for each dataset.

| **Pipeline Step** | **Best CH pipeline on average** | **Best DB pipeline on average** | **Best SIL pipeline on average** | **Best GSEA pipeline on average** |
| --- | --- | --- | --- | --- |
| **Filtering** | Default | Stringent | Stringent | Default |
| **Normalization** | Scran normalization | Seurat | Seurat | Scran normalization |
| **PCs used for clustering** | 30 | 30 | 30 | 15 |
| **Clustering resolution** | 0.1 | 0.1 | 0.1 | 1.2 |

**Table S3:** Parameters from top predicted pipeline for each metric.

| **Model** | **Random forest with dataset-pipeline features** | **Random forest with pipeline features only** | **Penalized linear regression including dataset-pipeline interactions** | **Penalized linear regression with pipeline features only** |
| --- | --- | --- | --- | --- |
| **CH** | 0.295 | 0.264 | 0.296 | 0.274 |
| **DB** | 0.266 | 0.193 | 0.227 | 0.137 |
| **SIL** | 0.130 | 0.105 | 0.0161 | 0.0794 |
| **GSEA** | 0.227 | 0.194 | 0.146 | ​​0.167 |

**Table S4:** Median correlation between predictions and observed metric values on test datasets across all models.

| **EBI ID** | **Organism part** | **Number of cells** |
| --- | --- | --- |
| E-CURD-79 | thymus | 119,540 |
| E-GEOD-139324 | blood | 157,689 |
| E-MTAB-8060 | embryo | 138,041 |
| E-MTAB-8207 | blood | 141,921 |
| E-MTAB-8894 | fetal straitum | 110,882 |

**Table S5:** Datasets with >100,000 cells used in analysis.

| **Models compared** | **RF and LR with interaction terms** | **RF and LR with pipeline features only** |
| --- | --- | --- |
| **Concordance with true metric values** | 0.844 | 0.842 |
| **Concordance with ARI** | 0.559 | 0.942 |

**Table S6:** Correlation between dataset feature-predictive power associations for RF and LR models with interaction features and pipeline features only. Predictive power quantified by concordance with true metric values and concordance with ARI.

| **Name** | **Description** | **Feature encoding for ML models** |
| --- | --- | --- |
| - Filtering:   - Lenient   - Default   - Stringent |  | Factor variable |
| - Normalization:   - Seurat   - Scran   - sctransform |  | Factor variable |
| - Dims:   - 10   - 15   - 20   - 30 | Dimensionality of PCA reduction prior to nearest neighbour selection for clustering | Numeric |
| - Resolution:   - 0.1   - 0.2   - 0.3   - 0.5   - 0.8   - 1.0   - 1.2   - 2 | Resolution of Seurat clustering | Numeric |

**Table S7:** Features computed for each pipeline.
